# Novel triangle tip‐jet knife increases efficiency in peroral endoscopic myotomy for achalasia

**DOI:** 10.1002/jgh3.12638

**Published:** 2021-09-06

**Authors:** Douglas Motomura, Simon Hew, Robert Bechara

**Affiliations:** ^1^ Gastroenterology Department Kingston Health Sciences Centre Kingston Ontario Canada; ^2^ Department of Gastoenterology Monash Health Melbourne Victoria Australia

**Keywords:** achalasia, esophageal motility, gastroenterology, peroral endoscopic myotomy

## Abstract

**Background and Aim:**

Peroral endoscopic myotomy (POEM) is performed globally for the treatment of achalasia. A newly available endoscopic knife, the triangle tip‐jet (TTJ) (Olympus Triangle TipKnife‐J, KD‐645L), has the capability of knife dissection along with submucosal injection. We aim to present our experience with the TTJ knife in comparison to the conventional TT knife in POEM, with a focus on procedural characteristics including time, efficiency, and the number of instrument exchanges.

**Methods:**

All patients with achalasia who underwent POEM between March 2016 and March 2020 at a single tertiary academic center were included in the retrospective cohort. Demographic, procedural, and outcomes data were compared.

**Results:**

Ninety‐two procedures, 48 using the TT knife, and 44 with the TTJ knife were analyzed. Demographic data were similar. Procedure time was reduced using the TTJ knife (87 *vs* 61 min, *P* = <0.001) despite similar myotomy lengths (16.5 *vs* 15.2 cm, *P* = 0.09). Efficiency was increased in the TTJ group (5.5 *vs* 4.3 min/cm of procedure, *P* = 0.005). The number of instrument exchanges (16.7–1.7, *P* = <0.001) and usage of coagulation forceps decreased (1.7–0.5, *P* = <0.001). There was no difference in the procedural difficulty (POEM difficulty score [PDS] 2.2 *vs* 2.4, *P* = 0.4). Patients with higher procedural difficulty saw a greater improvement in procedural outcomes. Procedural success was high in both groups (96% *vs* 100%, *P* = 0.2). No serious adverse events were reported.

**Conclusions:**

The use of the TTJ knife increases efficiency during POEM for Achalasia. The total procedure time is decreased by 28–41%, and procedural efficiency is increased by 22–34%.

## Graphical abstract

A novel triangle tip knife integrates injection and dissection function, decreasing the number of instrument exchanges required, leading to significantly shortened procedure times. We compare our experience using this new tool against the conventional knife.

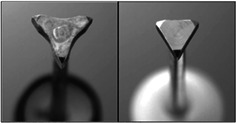

## Background

Achalasia is an esophageal motility disorder, characterized by incomplete lower esophageal sphincter (LES) relaxation and loss of normal esophageal peristalsis. Although the exact etiology of achalasia remains an area of active research, there is known to be a loss of inhibitory nitrous oxide neurons near the LES, and treatments have thus aimed to relieve the tonicity in this area and more proximally.^1^

The treatment of achalasia and spastic esophageal disorders has evolved significantly. Classically, the endoscopic management of achalasia consisted of botox injections and pneumatic dilations. The mainstay of surgical management is Heller myotomy. However, in 2010, a case series published by Inoue et al. described a technique of endoscopic myotomy.^2^ By creating a submucosal tunnel in the distal esophagus, the circular muscle bundle of the LES can be accessed and dissected. A meta‐analysis of non‐randomized studies has shown peroral endoscopic myotomy (POEM) to be at least as effective for the treatment of achalasia as compared to Heller myotomy.^3^ A recently published randomized non‐inferiority trial showed similar clinical outcomes between POEM and Heller myotomy.^4^ Randomized trials have also shown benefit *versus* repeat pneumatic dilations.^5^ Due to high efficacy and favorable safety profile,^6^ the popularity of POEM is expected to continue to grow in the coming years.

The original POEM protocol used the conventional triangle tip (TT) knife (KD‐640L; Olympus).^2^ The shape was selected to facilitate cutting in all directions without the need for rotation. One of the main limitations of the conventional knife is the need to exchange the instrument for an injection needle to expand the submucosa with saline and indigo carmine. Recently, an updated design was introduced in the form of a TT knife equipped with a built‐in water jet nozzle (Triangle Tip‐Jet, KD‐645L; Olympus). The injection function can be controlled via hand injection by an assistant or via foot pedal controlled by the main operator. This new design should theoretically reduce procedural time and the number of instrument exchanges, thereby leading to an increase in efficiency. To our knowledge, there is no North American data comparing the experience of conventional TT knife to triangle tip‐jet (TTJ).

## Methods

### Case selection

All patients with achalasia who underwent POEM between March 2016 and March 2020 at a single academic center in Ontario, Canada were included in the retrospective cohort. Diagnoses were made with a mixture of manometry, endoscopy, and radiographic findings. There were no prespecified exclusion criteria. Informed consent for both procedure and inclusion in future research was provided by patients before inclusion.

### Procedural technique

All cases were performed by one experienced operator. The POEM operator (R.B.) underwent a formal 1‐year fellowship at Showa University, Digestive Diseases Center. R.B. had experienced over 350 cases as an assistant or partial operator and performed over 60 independent cases prior to data collection. All procedures are performed following endotracheal intubation. Orientation is established using landmarks of the spine, trachea, left main bronchus, and aortic arch. The posterior approach was preferred, and thus the mucosal incision is performed in the 5 o'clock position after submucosal injection. A submucosal tunnel is created and extended toward the gastroesophageal junction. After the creation of the tunnel, the circular muscle bundles are identified, and selective myotomy is performed. The mucosal entry site is then closed with clips. Full technical details can be found in previous publications.^7^

**Figure 1:**
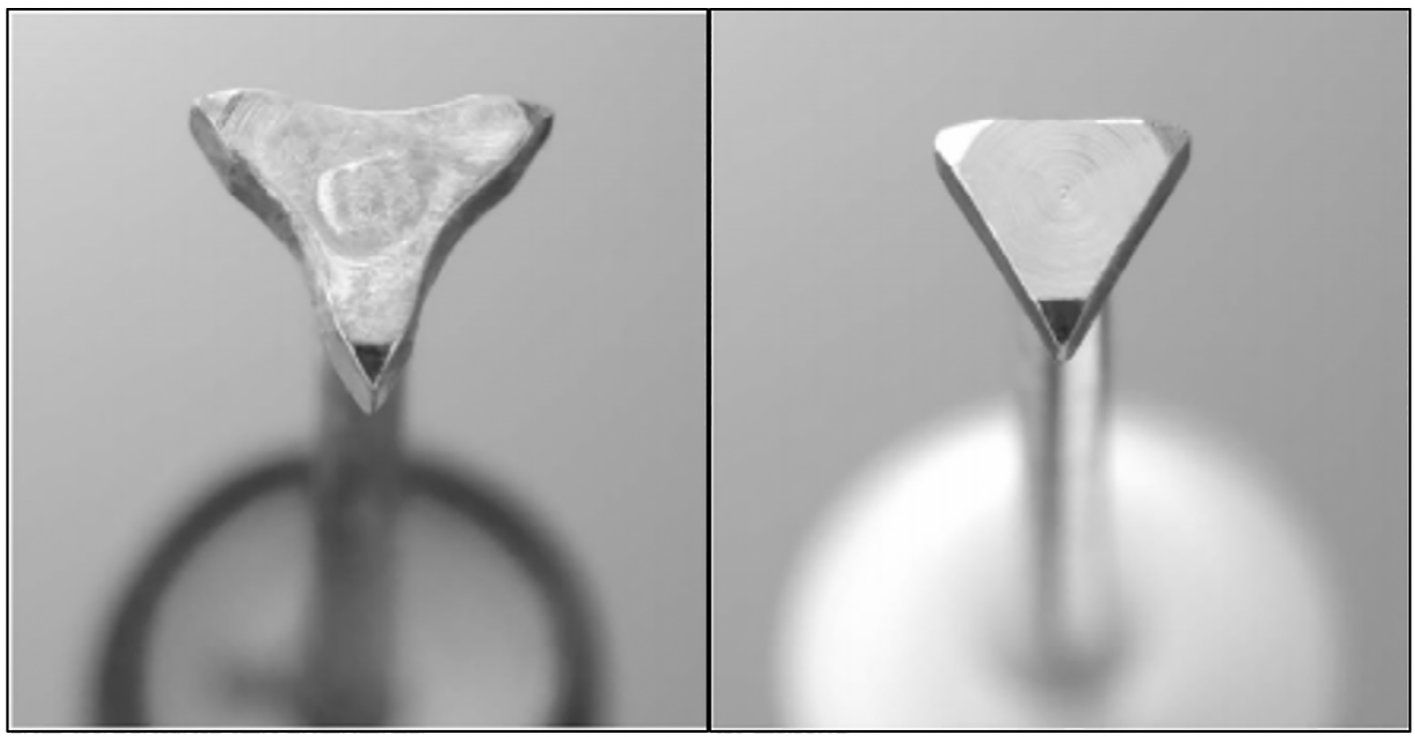
Conventional triangle tip (TT) knife (left). Triangle tip‐jet (TTJ) knife (right).

**Figure 2:**
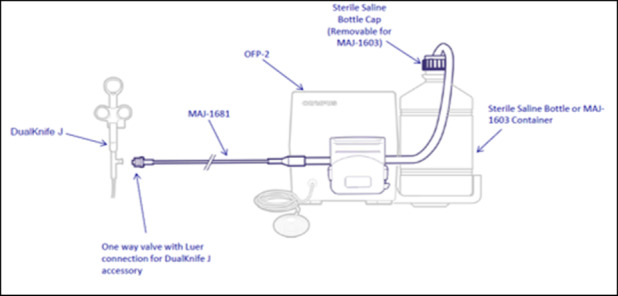
Foot pump setup used with triangle tip‐jet (TTJ) or DualKnife J (pictured). This allows for operator‐controlled injection through the instrument.

The conventional group underwent POEM using the TT knife (KD‐640L; Olympus). The TT cutting knife length is 4.5 mm with a diameter of 0.4 mm. The triangle tip head has a 0.7 mm radius and a thickness of 0.4 mm. The TTJ group underwent POEM using the TTJ knife (KD‐645L; Olympus). The cutting knife length is similar, with a length of 4.5 mm and a diameter of 0.4 mm. The triangle tip head is slightly smaller, with a radius of 0.4 mm, and a thickness of 0.3 mm. (Fig. 1, Olympus, unpublished). The injection function of the TTJ knife can be manually controlled via hand injection, or otherwise attached to a foot pedal system to be controlled by the main operator (Fig. 2).

### Data

Demographic information was collected on all patients analyzed, which included age, sex, body mass index (BMI), American Society of Anesthesiologists (ASA) score, achalasia subtype, previous treatment, pre‐procedure Eckardt score, and symptom duration. The Eckardt score consists of four cardinal symptoms including dysphagia, weight loss, regurgitation, and chest pain. Each component is graded from 0 to 3 based on the reported frequency of the symptom, with 0 representing “never” and 3 representing “every meal.” The total score is additive and thus ranges from 0 to 12.^8^

Our primary outcome of interest was procedure time, defined as the start of the creation of the submucosal injection to the final clip application. Additional procedural data including the difficulty of the procedure, the efficiency of myotomy, the number of instrument exchanges, and usage of coagulation forceps were reported as secondary outcomes. Efficiency is defined as the total procedural time over the total length of myotomy during the procedure (min/cm). Length of myotomy was selected as a surrogate that encompasses both submucosal tunneling as well as the selective myotomy itself.

The procedural difficulty was described using the POEM difficulty score (PDS), which has been used previously to quantify the difficulty of complex procedures.^9^ The PDS takes into account five factors involved in procedural difficulty, summarized by the acronym “FOODS”: **f**ibrosis, **o**ozing (hemorrhage), **o**rientation, **d**istention of the submucosal tunnel, and **s**pastic contractions. Each factor is assigned a value between 0 and 2, with 2 representing the most difficulty. The total score is calculated additively, with a maximum score of 10. Higher difficulty scores have been shown to correlate with lower efficiency.^9^

We also noted clinical outcomes, including clinical success, defined as post‐POEM Eckardt score of ≤3 at 3 months, and sustained improvement at the most recent follow‐up.

### Statistical analysis

Demographic, procedural, and clinical data are presented as percentages for categorical data and mean with standard deviation for continuous data. These were analyzed with two‐tailed t testing. Categorical data were analyzed via chi‐square testing. Ninety‐five percent confidence intervals are provided when describing the differences of means. Results are presented with *P* values with alpha at 0.05. Statistical analysis was performed using Microsoft Excel with the Data Analysis Toolpak.

## Results

A total of 92 procedures were performed during the study period. Forty‐eight were performed with the conventional TT knife, and the remaining 44 were performed with the TTJ. Baseline patient demographics are presented in Table 1. Despite non‐randomization, there were no significant differences encountered between the two groups of patients.

**Table 1:** Baseline demographics and characteristics

		Conventional (*n* = 48)	TTJ (*n* = 44)	
Age		54.3 ± 18.2	51.2 ± 21.2	*P* = 0.45
Sex	M	26 (54.2%)	25 (56.8%)	*P* = 0.80
	F	22 (44.8%)	19 (43.2%)	
BMI		28.0 ± 7.3	25.6 ± 6.1	*P* = 0.09
ASA score		2.6 ± 0.7	2.2 ± 0.7	*P* = 0.06
Achalasia subtype	1	10 (20.8%)	6 (13.6%)	*P* = 0.08
	2	21 (43.8%)	26 (59.1%)	
	3	17 (35.4%)	9 (20.5%)	
	Other	0 (0%)	3 (6.8%)	
Prior treatment		24 (50.0%)	16 (36.4%)	*P* = 0.19
Eckardt score		7.6 ± 2.0	6.9 ± 2.3	*P* = 0.09

Continuous data presented as means ± SD. Conventional: triangle tip knife.

ASA, American Society of Anesthesiologists; BMI, body mass index; TTJ, triangle tip with jet nozzle.

The main procedural outcomes are presented in Table 2. All procedures achieved technical success. Despite no significant difference in the total length of myotomy performed in the two groups (16.5 *vs* 15.2 cm, *P* = NS), the mean procedure time was shortened from 87 to 61 min (31% reduction, *P* = <0.001) when comparing the TTJ to the conventional group. The mean procedural efficiency was improved from 5.5 to 4.3 min/cm (25% improvement, *P* = 0.005). The mean number of instrument exchanges was reduced from 16.7 to 1.7 (90% reduction, *P* = <0.001), and usage of coagulation forceps for hemostasis was decreased from 1.7 to 0.5 (59% reduction, *P* = <0.001). There was no significant difference in the difficulty of each POEM, as described using the PDS (2.2 *vs* 2.4, *P* = NS). There were no major adverse events reported for either group (Clavien–Dindo grade IIIb‐V^10^). One patient in the TT group had a 1 cm epithelial hematoma (Clavien–Dindo grade I) visualized on the post‐POEM day 1 endoscopy. The patient was asymptomatic, and no intervention was required.

**Table 2:** Procedural outcomes

	Conventional (*n* = 48)	TTJ (*n* = 44)	Mean difference [95% CI]	
Total myotomy length (cm)	16.5 ± 3.2	15.2 ± 4.3	1.3 [−0.2, 2.9]	*P* = 0.09
Total procedure time (min)	87.0 ± 28.9	61.2 ± 19.0	26.8 [16.9, 36.7]	*P* = <0.001
Procedural efficiency (min/cm)	5.5 ± 2.3	4.3 ± 1.8	1.2 [0.4, 2.1]	*P* = 0.005
POEM difficulty score	2.2 ± 1.5	2.4 ± 1.8	−0.3 [−1.0, 0.4]	*P* = 0.4
Instrument exchanges (#)	16.7 ± 7.0	1.7 ± 1.8	15.0 [12.8, 17.1]	*P* = <0.001
Use of coagulation forceps (#)	1.7 ± 1.5	0.5 ± 0.7	1.1 [0.6, 1.6]	*P* = <0.001
Adverse events	0	0	n/a	n/a

Continuous data presented as means ± SD. Conventional: triangle tip knife.

TTJ, triangle tip with jet nozzle.

The differences in procedural time, procedural efficiency, and instrument exchanges were sustained when stratifying for PDS (Table 3). In the high PDS group, the procedural time was reduced from 117.2 to 68.4 min (41% reduction, *P* = <0.001), the improvement in procedural efficiency was increased from 8.0 to 5.2 min/cm (34% improvement, *P* = 0.001) and the number of instrument exchanges by from 19.8 to 1.4 (90% reduction, *P* = <0.001). The myotomy lengths remained comparable (15.2 cm *vs* 14.4 cm, *P* = 0.54). In the low PDS group (PDS ≤2), operative time was reduced from 74.7 to 53.9 min (28% reduction, *P* = <0.001) and efficiency increased from 4.4 to 3.4 min/cm (22% increase, *P* = <0.001).

**Table 3:** Procedural outcomes (difficulty subgroup)

PDS ≥3	Conventional (*n* = 15)	TTJ (*n* = 22)	
Total myotomy length (cm)	15.2 ± 3.5	14.4 ± 4.7	*P* = 0.54
Total procedure time (min)	117.2 ± 29.8	68.4 ± 21.5	*P* = <0.001
Procedural efficiency (min/cm)	8.0 ± 2.4	5.2 ± 2.0	*P* = 0.001
Instrument exchanges (#)	19.8 ± 5.7	1.4 ± 1.5	*P* = <0.001
Use of coagulation forceps (#)	1.8 ± 1.6	0.5 ± 0.7	*P* = 0.02

PDS ≤2	Conventional (*n* = 33)	TTJ (*n* = 22)	
Total myotomy length (cm)	17.1 ± 2.9	16 ± 3.8	*P* = 0.25
Total procedure time (min)	74.7 ± 16.0	53.9 ± 12.3	*P* = <0.001
Procedural efficiency (min/cm)	4.4 ± 1.1	3.4 ± 0.7	*P* = <0.001
Instrument exchanges (#)	15.4 ± 7.2	2.0 ± 2.1	*P* = <0.001
Use of coagulation forceps (#)	1.6 ± 1.5	0.6 ± 0.7	*P* = 0.002

Continuous data presented as means ± SD. Conventional: triangle tip knife.

TTJ, triangle tip with jet nozzle; PDS, POEM difficulty score; POEM, peroral endoscopic myotomy.

Clinical outcomes were available for all patients (Table 4). Post‐procedure Eckardt scores achieved a mean value of 1.2 in the conventional group and 1.1 in the TTJ group (*P* = 0.50, NS). This represented an improvement in score of 6.4 points and 5.9 points respectively from pre‐procedure. Clinical success was achieved in 46 of 48 patients in the TT group and 38 of 38 patients in the TTJ group (*P* = 0.2, NS). The duration of follow up was longer in the TT group (median 24 *vs* 8.5 months).

**Table 4:** Clinical outcomes

	Conventional (*n* = 48)	TTJ (*n* = 44)	
Post‐procedure Eckardt score	1.2 ± 1.4	1.1 ± 0.8	*P* = 0.50
Post‐procedure Eckardt ≤3 (%)	46 (96%)	44^†^ (100%)	*P* = 0.2
Follow‐up duration (months)	24.3 ± 8.0	8.7 ± 4.4	*P* = <0.001

^†^ One patient had follow up before the 3‐month mark.

Continuous data presented as means ± SD. Conventional: triangle tip knife.

TTJ, triangle tip with jet nozzle.

## Discussion

Our data indicate that there is a significant reduction in total procedure time and an increase in procedural efficiency using the TTJ knife. This is hypothesized to be driven by the reduction in the number of instrument changes during the procedure (from a mean of 16.7 to 1.7 exchanges per procedure). The standard TT knife requires the complete removal of the needle knife instrument and insertion of the injection needle down the working channel when performing the submucosal injection. Multiple injections are used to elucidate the submucosa throughout the tunneling phase. The TTJ knife allows this injection to be performed without removal of the needle knife, via manual injection from an assistant, or by a foot pedal, as was standard in our cohort. Although efficiency in our series is reported as total procedural time per cm of myotomy, most of the time‐saving likely stems from the tunneling phase. The length of myotomy was chosen for representation as it is more easily measured, and also dictates the length of the submucosal tunnel.

Complex procedures often require more instrument exchanges to maintain a safe and efficient submucosal tunnel (19.8 exchanges in the high PDS TT group *vs* 15.4 exchanges in the low PDS TT group). Thus, the improvement was even more pronounced in the more difficult POEM procedures (41% reduction in high PDS procedural time *vs* 28% reduction in low PDS), leading to relative increased efficiency (34% increase in high PDS *vs* 22% increase in low PDS).

Although there is no difference in the hemostatic ability of the TTJ compared with the TT knife, the usage of coagulation graspers was significantly decreased (mean of 1.7–0.5 uses per procedure). This is hypothesized to be a byproduct of the jet function in the TTJ. By allowing for easier and more frequent injections, the submucosal cushion can be maintained more reliably. This can improve the visualization of penetrating vessels and prevent inadvertent severing during the tunneling phase, particularly at the gastroesophageal junction.

There were no differences in clinical success, as defined by Eckardt score post‐procedure of ≤3. (96% *vs* 100%, *P* = NS). One patient in the TT group had nonresponse after initial POEM and underwent a second POEM using an anterior approach, with subsequent Eckardt score of 1. A second patient in the TT group had maintained clinical success for 4 years before the recurrence of symptoms, and Eckardt score increased to 6. This patient underwent repeat POEM with the anterior approach, with subsequent Eckardt score of 2. All patients in the TTJ group had clinical success at the time of the most recent follow‐up.

Previous studies have shown usage of conventional TT knives as a factor predicting longer procedural time in multivariate analysis when compared with HybridKnife,^11^ which is an ERBE branded product used for POEM and endoscopic submucosal dissection. Similar to the TTJ, the HybridKnife has the advantage of integrated water jet into the tip. The HybridKnife has similar cutting length as the TTJ, with a 5 mm knife and a variety of head shapes.^12^

However, only one previous study has described the experience with the TTJ knife.^13^ Nabi et al. showed a significant reduction in operative time with the TTJ knife compared with the TT knife in a single‐center analysis comprising 193 patients in Hyderabad, India. They were able to demonstrate an improvement in operating time from 66 to 54 min. Similarly, they showed a significant decrease in the usage of instrument exchanges (10.5 exchanges/procedure to 2.9 exchanges/procedure) and the use of coagulation forceps (4.7 uses/procedure to 2.9 uses/procedure).

We aimed to build upon this study by presenting North American data, as well as presenting a comparison of procedural efficiency to account for potential differences in tunnel and myotomy lengths between procedures. Our data also demonstrate that this effect is even more pronounced in the more difficult cases, which inevitably tend to have longer operative times.

**Figure 3:**
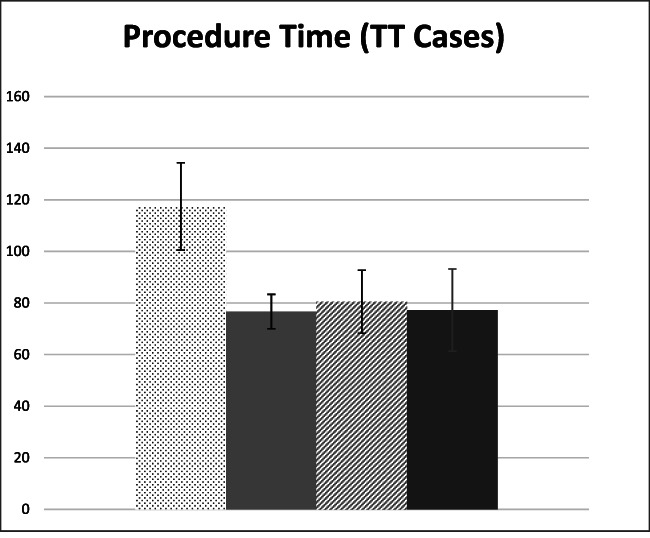
TT (triangle tip). Q1—First 25% of cases. Q2—second 25% of cases. Q3—third 25% of cases. Q4—last 25% of cases. Error bars denote 95% confidence interval (CI). 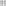, Q1; 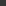, Q2; 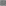, Q3; 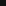, Q4.

**Figure 4:**
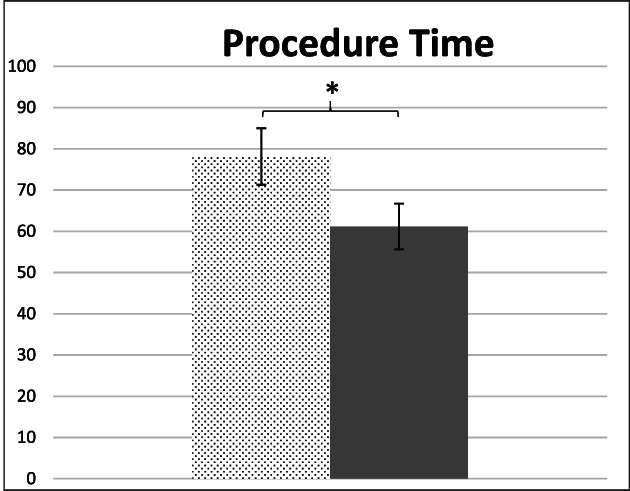
TT: triangle tip (conventional) TTJ: triangle tip‐jet *P = <0.01. 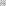, TT (excluded Q1); 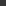, TTJ.

One limitation of this non‐randomized retrospective study is the study period. The conventional TT knife was utilized until case number 52, after which all cases were performed with the TTJ knife. As such, the TTJ cases were performed more recently. Thus, the follow‐up period for the TT group is longer than the TTJ group. Moreover, this raises the question of whether progressive operator experience may have contributed to the improved procedural outcomes. However, previously published studies have noted that procedural time and efficiency begin to plateau after the first 20 cases,^14^ and “mastery” can be achieved after 60 cases.^15^ The sole operator (R.B.) had extensive experience with POEM prior to data collection, including over 60 cases as an independent operator and over 300 cases as an assistant/partial operator prior to data collection. Further analysis shows the first quarter of TT cases (12 cases totally) were significantly longer than quarters two to four (Fig. 3). Given the rapid and sustained plateau, we think it is unlikely that operator performance accounted for this improvement; however, other periprocedural factors were likely at play. These were among the first third space procedures performed at our center, and were used to train procedural assistants(total of six rotating assistants) in the setup, handling, and usage of the specialized tools. To try to mitigate this learning curve, we repeated the analysis excluding the first quarter of cases (Fig. 4). The results remained significant (78.1 *vs* 62.1 min, *P* = <0.001).

A second limitation is inherent in the single‐center, single‐operator nature of our investigation, which reduces the generalizability. As POEM continues to grow and evolve in Canada, we expect more local and national data to become available. Finally, although immediate procedural success and short‐term clinical outcomes were similar between the two groups, long‐term clinical outcomes will need to be followed to ensure no differences emerge, and the effects prove durable.

The optimization of procedural efficiency remains of significant importance in the current North American landscape of resource scarcity. A cost analysis including laparoscopic Heller myotomy, POEM, pneumatic dilations, and botulinum toxin injections demonstrated comparable costs between surgical or endoscopic myotomy over a 4‐year period at a single US center.^16^ The cost per cure for POEM begins at $12 120 at year 1, and decreases to $3030 at year 4. The cost per cure for laparoscopic Heller myotomy begins at $11 582 at year 1 and decreases to $2896 at year 4. We expect our local costs per cure for POEM to be even lower, given shorter hospital stays (mean 1.1 days) compared with the analysis performed by Miller et al. (mean of 3 days).

Further improvements in cost‐efficiency may aim to target reduced procedural time. Although the TTJ knife is sold at an increased price in comparison to the TT knife at the time of publication ($1750 *vs* $827, list prices in USD, unpublished, Olympus), the financial benefits can be demonstrated. A recent analysis of operating theater costs estimated the cost per minute of theater time to be approximately $37.45/min.^17^ With a mean difference of 26.8 min in procedural time between the TT and TTJ group, this translates to a savings of $1004 per case, greater than the cost difference between the instruments. Again, this effect is more pronounced in more difficult cases, with a mean difference of 48.8 min and potential savings of 1828$ per case in the PDS ≥3 subgroup. In addition to the direct cost–benefit, shorter procedural time potentially allows more procedures to be scheduled, reducing the persistent strain on the healthcare system.

Thus, TTJ is shown to decrease procedural time and increase procedural efficiency during POEM for achalasia in North America and can be considered for use as the standard knife during POEM. Our data suggest an improvement of procedural time of 28–41%, and an increase in procedural efficiency of 22–34%, with improvement being more pronounced in more difficult cases. There is no change in technical or clinical success. Although there is an additional upfront cost when compared with the conventional TT knife, cost‐efficiency is possible due to the significantly reduced operative time.
